# Lower Serum Calcium Levels Associated with Disrupted Sleep and Rest–Activity Rhythm in Shift Workers

**DOI:** 10.3390/nu14153021

**Published:** 2022-07-22

**Authors:** Yi-Seon Jeon, Seungyeong Yu, Chaeyeon Kim, Hyuk Joo Lee, In-Young Yoon, Tae Kim

**Affiliations:** 1 Department of Biomedical Science and Engineering, Gwangju Institute of Science and Technology, Gwangju 61005, Korea; 2sun98@kaist.ac.kr (Y.-S.J.); osos5918@gm.gist.ac.kr (S.Y.); rlacodus96@gist.ac.kr (C.K.); 2 Current affiliation: Department of Biological Sciences, Korea Advanced Institute of Science and Technology, Daejeon 34141, Korea; 3 Department of Public Medical Service, Seoul National University Bundang Hospital, Seongnam 13620, Korea; dekn11@hanmail.net; 4 Department of Psychiatry, Seoul National University Bundang Hospital, Seongnam 13620, Korea; iyoon@snu.ac.kr; 5 Department of Psychiatry, Seoul National University College of Medicine, Seoul 03080, Korea

**Keywords:** vitamin D, calcium, sleep, rest-activity rhythm, actigraphy, shift worker, non-shift worker

## Abstract

Vitamin D deficiency is prevalent in many developed countries, and several studies suggest that vitamin D plays an essential role in brain function. A recent study showed that vitamin D deficiency was closely associated with daytime sleepiness and shorter sleep time. The relationshipbetween vitamin D levels and calcium levels is well established, and calcium level regulates slow-wave sleep generation. It is conceivable that the sleep disturbance in vitamin D deficiency may be due to an altered calcium level. Nonetheless, calcium levels, sleep disturbances, and activity rhythms have not been investigated directly. Therefore, we hypothesized that calcium and vitamin D levels might be important in regulating sleep and activity rhythm, and we analyzed the correlation with calcium levels by actigraphy analysis. Interestingly, a negative correlation was found between calcium level and sleep latency, total sleep time, use of sleep medicine, and daytime dysfunction among shift workers. In contrast, non-shift workers showed a negative correlation between the calcium level and the circadian phase. These findings suggest that low serum calcium levels may disrupt sleep–wake control and rest–activity rhythm, even if they are within the normal range.

## 1. Introduction

During shift work, sleep–wake cycles are misaligned with the endogenous circadian system, causing metabolic disruption and negatively affecting overall health [1]. Prior studies suggest prolonged night shift working may increase the risk for sleep and gastrointestinal problems, as well as aberrant metabolic responses [2,3,4]. Moreover, the disturbances of circadian rhythm are associated with major diseases, such as cardiovascular diseases or cancer [5,6]. Since shift workers have the disadvantage of irregular sleep schedules, they are at remarkably high risk of developing sleep problems.

By exposing the skin to ultraviolet light, vitamin D is photosynthesized from sterols in the body. With a lack of moderate sun exposure, vitamin D deficiency has become more prevalent in the modern population, and its relationship with health problems was suggested [7]. On the other hand, it was suggested that night shift working may contribute to lower serum vitamin D levels [8,9]. It was plausible results because night shift workers may spend less time for sunlight exposure. Recently, there is a hypothesis that vitamin D may play a role in sleep regulation. Vitamin D receptors (VDRs) are expressed in brain regions related to sleep–wake regulation, including the bed nucleus in the stria terminalis, the thalamic reticular nucleus, the central nucleus of the amygdala, and the dorsal raphe nucleus [10,11,12,13]. In addition, A deficiency of vitamin D is also connected with the global prevalence of sleep disorders [14]. Recent studies have reported that vitamin D deficiency is linked to reduced sleep duration and lower efficiency [15,16,17]. Several studies have also shown that vitamin D supplements improve sleep quality, implying that an insufficient vitamin D intake may cause sleep problems [17,18]. In addition to sleep regulation, vitamin D can regulate the molecular clock [19], and the suprachiasmatic nucleus (SCN), the master clock of the circadian rhythm, contains a significant number of VDRs [20]. These studies suggest the possibility of a potential link between vitamin D and circadian rhythmic regulation.

Classic functions of vitamin D are regulating calcium transport in the intestine and mineralizing bones, which are essential for calcium homeostasis [21]. Calcium homeostasis is tightly controlled by the active form of vitamin D and parathyroid hormone [22]. Recent papers have reported that calcium is associated with sleep regulation, especially for non-rapid eye movement (NREM) sleep generation. Cortical slow-wave oscillations are mediated by calcium fluctuation and intracellular potentials in sleep. A synchronous and rhythmic alternation between depolarized and hyperpolarized membrane potentials makes up the main activity of cortex neurons during NREM sleep [23]. In addition, calcium signaling regulates the duration of NREM sleep by controlling the slow wave activity. [24]. Moreover, another study found that calcium level was associated with sleep latency and non-restorative sleep-in questionnaire assessment [25]. Taken together, calcium levels may be an important contributing factor to sleep–wake regulation. 

However, there is a lack of studies that investigated the inter-relationship among vitamin D, calcium, sleep–wake control, and rest–activity rhythm pattern, depending on working schedules. Therefore, we hypothesized that the vitamin D and calcium levels might be affected by the shift working and sought to investigate the relationship between activity-based parameters of sleep and rest–activity rhythm and vitamin D and calcium levels in shift and non-shift workers.

## 2. Materials and Methods

### 2.1. Subjects

**Figure 1:**
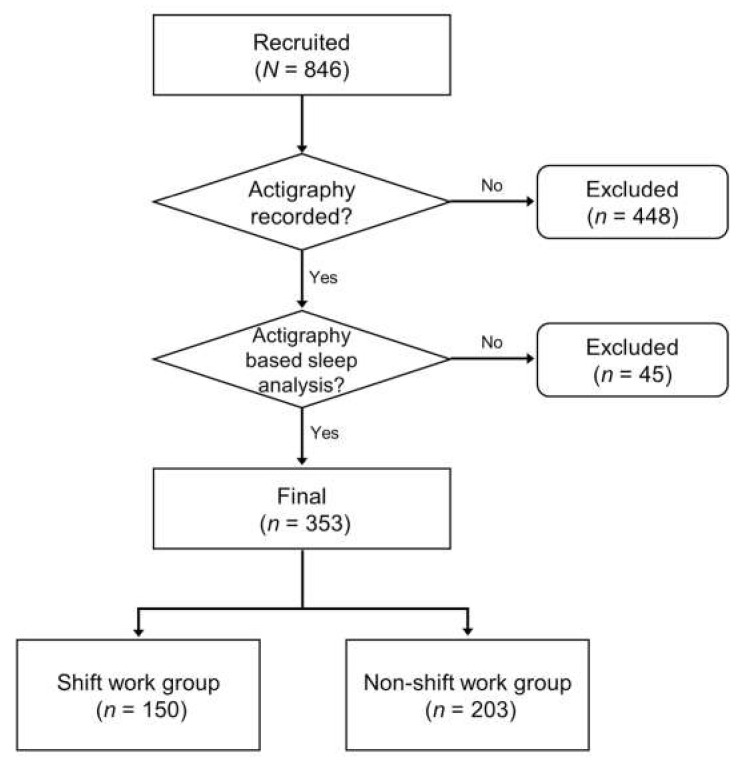
Flow chart of study design.

Data were collected from 846 employees from Seoul National University Bundang Hospital (SNUBH). This study population included 412 shift workers and 434 non-shift workers aged 20 to 65. The data were collected from November 2017 to January 2019. In this paper, “shift workers” work more than or equal to six-night shifts in a month (working schedules are 6:00 p.m.–8:00 a.m., 7:00 p.m.–7:00 a.m., or 10:00 p.m.–7:00 a.m.). Otherwise, “non-shift workers” refer to those who work fixed daytime schedules (working schedules are set from 9:00 a.m. to 6:00 p.m.) or have shift work schedules without night (working schedules from 7:00 a.m. to 3:00 p.m. or from 2:00 p.m. to 10:00 p.m.). Of the total subjects (*N* = 846), 398 subjects participated in actigraphy recordings for successive days. We selected the final analysis group (*n* = 353) with actigraphy data for at least seven days. This last group comprised 150 shift workers and 203 non-shift workers (Figure 1). The data collected from this final group included actigraphy-based sleep and rest–activity rhythm parameters, clinical evaluation results, demographic information, and serum analysis.

### 2.2. Demographic and Clinical Characteristics

From SNUBH, self-report questionnaires were used to collect clinical information and assess subjective sleep quality. These evaluation scales provide information regarding subjective sleep quality, chronotypes, depressive and anxiety symptoms, fatigue, and resilience. For evaluating sleep quality, the Pittsburgh sleep quality index (PSQI) was used, in which a total PSQI score >5 indicates low subjective sleep quality [26]. The Epworth sleepiness scale (ESS) was used to assess daytime sleepiness, and a higher ESS score indicates more severe daytime sleepiness [27]. Morningness-eveningness questionnaire (MEQ) score refers to morning preference, while a lower MEQ score indicates evening preference [28]. The hospital anxiety and depression scale (HADS) suggests a higher anxiety and depression status with a higher score [29]. The fatigue severity scale (FSS) was also adopted to evaluate subjective fatigue status [30]. The Connor–Davidson resilience scale (CD-RISC) was used to evaluate psychological resilience. CD-RISC scores range from 0 to 100, and higher scores indicate higher stability, such as hardiness or perceived stress [31].

Demographic characteristics and blood parameters were also obtained. These included age, sex, body-mass index (BMI), marital status (married = 1, single = 0), an education level (based on college), current smoking status, coffee consumption, drinking habits, physical activity, medication use, medical comorbidities, and serum levels of metabolites such as vitamin D and calcium. For the medication use, we checked the current medication use status by yes/no question. Although various types of medication have been reported, we used nominal data for the analysis. Blood samples were collected and processed during the daytime (between 10:00 a.m. to 2:00 p.m.) at the testing institute (Seoul Clinical Laboratories, Seoul, Korea) in all seasons from November 2017 to January 2019. A chemiluminescent immunoassay method was used for the measurement of 25(OH)D in serum.

### 2.3. Actigraphy-Based Sleep Analysis

An actigraph (wGT3X-BT, ActiGraph, Pensacola, FL, USA) is used to monitor the human sleep–wake cycle. Actigraphs were worn on the non-dominant wrists of subjects selected for the final group, and activities were recorded for 14 consecutive days (1-min epoch). From actigraphy recorded data and the diary of each subject, sleep parameters were calculated using software (Actilife6, ActiGraph, Pensacola, FL, USA). The Cole-Kripke sleep algorithm determined sleep and wake states [32]. With ActiLife6 software, data were analyzed to the following parameters: total sleep time (TST), time in bed (TIB), sleep efficiency (SE, TST/TIB), sleep latency (SL, time in minutes to fall asleep), and wake after sleep onset (WASO, time in minutes from sleep onset to awake).

### 2.4. Cosinor Analysis

**Figure 2:**
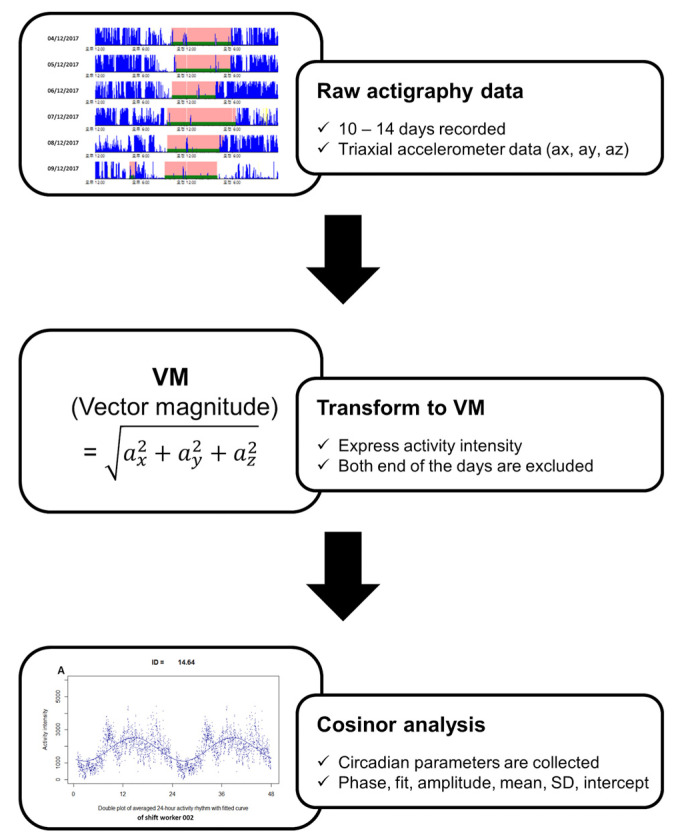
Flowchart of cosinor analysis process to collect actigraphy-based activity parameters. Raw actigraphy data were transformed as vector magnitude (VM) values, and rest–activity rhythm parameters were collected by cosinor analysis. This analysis was performed using R software.

Cosinor analysis is a periodic regression with a fixed period [33]. This analysis was used for rest–activity data, which is circadian periodic with a cycle of 24 h. The equation of regression model can be written as Y (t) = M + Acos (2πt/τ + φ) + e (t). Here, M denotes the midline estimating statistic of rhythm (MESOR, mean value of oscillation), A is the amplitude, φ is the acrophase (the time period at which the peak in each cycle), τ is the period (duration of one cycle), and e (t) is the error term. This analysis was performed using R software to collect parameters from the raw actigraphy data. The Cosinor analysis was conducted as follows. Raw actigraphy data were transformed to 24-h average vector magnitude (VM) values, indicating daily activity intensity. As the results of this analysis, six rest–activity parameters could be estimated, namely, phase, mean, amplitude, fit, SD, and intercept (Figure 2). Phase (acrophase) means the time of day of the peak of the curve, and a later time suggests more phase delay. Fit indicates the value of the correlation of the fit. Amplitude is the height of the rhythm, which equals the minimum activity’s subtraction from the maximum value. A lower amplitude suggests a dampened rest–activity rhythm. The mean is the MESOR, and lower values indicate less activity and rhythmicity. The intercept is the activity intensity value of the cosinor function when the time is 0. SD is the standard deviation, which means how much the dataset is scattered around its mean. Thus, a lower SD of the amplitude means a lower amplitude value. 

### 2.5. Statistical Analysis

Origin software (OriginLab, Northampton, MA, USA) and R software, version 4.0.0 (https://www.r-project.org/) were used for statistical analysis. To compare the demographic information of shift and non-shift workers, an independent *t*-test and chi-square test by SPSS software was performed for continuous and categorical variables, respectively. We used the Origin software for a paired-sample students’ *t*-test to evaluate sleep and rest–activity rhythm parameter differences according to the working status. Using R software, Pearson’s correlation test was adopted to investigate the correlation between calcium and vitamin D levels and parameters for sleep and rest–activity rhythm. A two-tailed *p*-value of <0.05 was considered statistically significant for all statistical analyses, expressed as * *p* < 0.05 or ** *p* < 0.01, respectively.

## 3. Results

Population and demographic information were collected (Table 1). Table 1 compares the demographic data of shift and non-shift workers in the final group (Figure 1, Level C). As a demographic comparison between shift workers and non-shift workers, shift workers were significantly younger (*p* < 0.001) and had lower BMI scores (*p* < 0.001) and higher education levels (*p* < 0.001, 93.3% vs. 75.3%). Moreover, a smaller proportion of shift workers were married or smokers (*p* < 0.001, both). In addition, shift workers showed lower use of medication (*p* = 0.058). Finally, shift worker reported higher consumption of alcohol and more physical activity and had lower caffeine consumption and lower medical comorbidities, but there were no statistical significance or trend.

**Figure 3:**
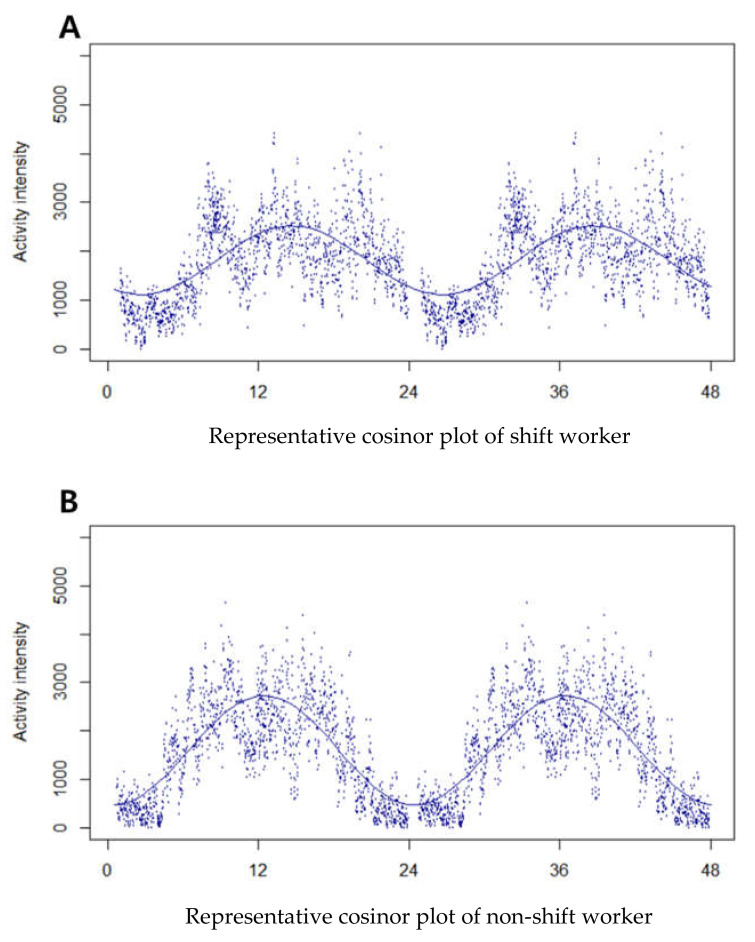
Representative cosinor plots of shift and non-shift worker. Activity intensity means vector magnitude values of activity recorded by actigraph. ID indicates phase (acrophase), and the x-axis is responding time. (**A**) A double plot of averaged 24-h activity rhythm with the fitted curve of shift worker 002. (**B**) A double plot of averaged 24-h activity rhythm with the fitted curve of non-shift worker 012.

Sleep, rest–activity rhythmic, and clinical information were collected (Table 2). Table 2 compares the clinical and actigraphy-based sleep and rest–activity parameters of shift and non-shift workers in the final group. Shift workers have lower serum calcium levels (shift workers: 9.31 ± 0.03, 8.3 to 10.5 mg/dL vs. non-shift workers: 9.38 ± 0.02, 8.6 to 10.7 mg/dL), not vitamin D (shift workers: 13.45 ± 0.46, 4 to 45.6 ng/mL vs. non-shift workers: 14.58 ± 0.59, 4.4 to 66 ng/mL). However, 10 and 7 subjects in shift and non-shift groups, respectively, showed below the normal range of serum calcium level. Moreover, 15 and 1 subjects in shift and non-shift groups, respectively, showed normal vitamin D levels. The distribution of calcium deficiency in shift and non-shift groups was not different (*p* > 0.05, chi-square test) and that of normal vitamin D levels were lower in the shift worker group (*p* < 0.05, Fisher’s exact test). Further analysis of vitamin D levels after excluding vitamin D supplementation cases also showed lack of difference in vitamin D levels between the two groups. There was no difference in the comparison of vitamin D levels between groups, but the ratio of normal level (>30 ng/mL) was significantly lower in shift workers. As expected, the shift workers showed poorer sleep quality and lower rest–activity rhythmicity in this study. Based on actigraphy results, shift workers statistically had lower total sleep time, longer WASO, and lower sleep efficiency (*p* < 0.001 for each variable). Shift workers also showed significantly disrupted rest–activity rhythm parameters, such as delayed phase, lower fit, and lower amplitude, which means lower rest–activity rhythmicity than non-shift workers (Figure 3). Based on the clinical evaluation parameters, shift workers reported that they tended to prefer evening to morning (lower MEQ, *p* < 0.001), severe daytime sleepiness (higher ESS, *p* < 0.001), anxiety/depression (higher HADS, *p* < 0.05), poor subjective sleep quality (higher PSQI, *p* < 0.001), and higher fatigue levels (higher FSS, *p* < 0.001) than non-shift workers.

**Figure 4:**
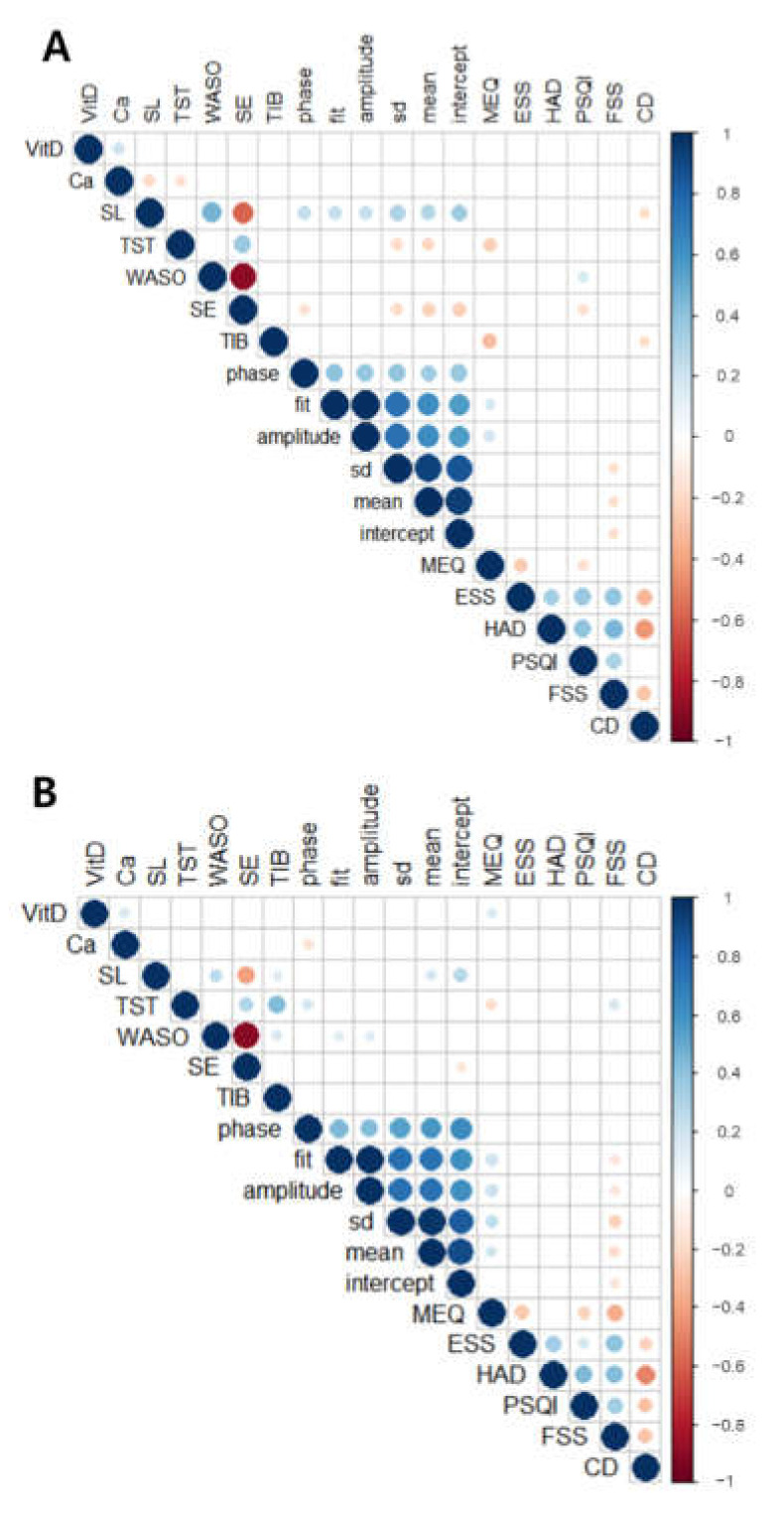
Correlation plots of each parameter from shift and non-shift workers. (**A**) Correlation plot of shift workers (*n* = 150) and (**B**) correlation plot of non-shift workers (*n* = 203). Only a statistically significant correlation (*p* < 0.05) is shown in these plots. The color and size of each circle reflect the value of Pearson’s correlation coefficient r (blue: positive and red: negative).

The impact of serum calcium level on actigraphy-based sleep parameters was also assessed. In the correlation analyses, serum calcium level was significantly correlated with parameters for sleep and rest-activity rhythm (Figure 4). Figure 4 presents the correlation plot for shift and non-shift workers. Among the variables, only statistically significant correlations were expressed. Both groups showed a positive correlation between vitamin D and calcium (r = 0.20, *p* = 0.01; r = 0.15, *p* = 0.02, respectively), represented as a blue circle. Moreover, with the calcium row, there are two significant negative correlations in shift workers (sleep latency and total sleep time) and one in non-shift workers (phase), which are represented as an orange circle.

**Figure 5:**
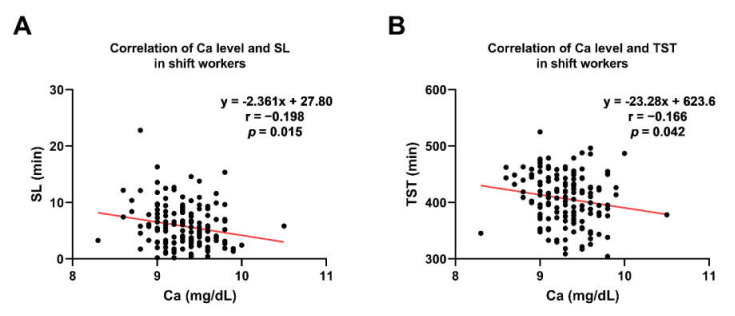
There are significant correlations between Ca levels, sleep latency, and total sleep time in shift workers. (**A**) Ca levels showed a negative correlation with sleep latency in the shift workers. (**B**) Ca levels negatively correlated with total sleep time in the shift workers. Red lines represent trend lines by linear regression. *Abbreviations*: SL, sleep latency; TST, total sleep time; R, Pearson’s correlation coefficient.

**Figure 6:**
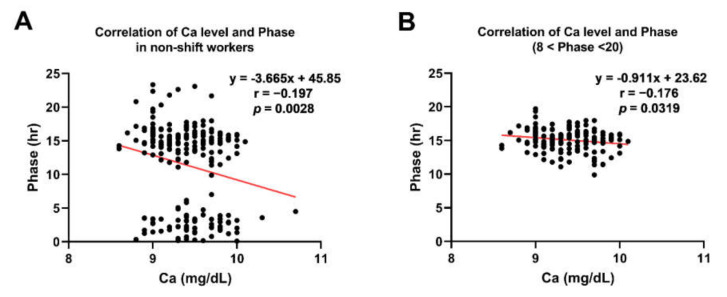
Significant correlations between Ca levels and phase in non-shift workers. In daytime non-shift workers, there was no meaningful relationship between sleep parameters. (**A**) Ca levels negatively correlated with circadian phase in non-shift workers. (**B**) In order to confirm correlation, the significance is also checked in the subjects with the phase between 8 and 20. Red lines represent trend lines by linear regression. *Abbreviations*: SL, sleep latency; TST, total sleep time; R, Pearson’s correlation coefficient.

In the actigraphy-based analysis, the calcium level was negatively correlated with sleep latency and total sleep time in shift workers (Figure 5A,B; r = −0.20, *p* = 0.02; r = −0.17, *p* = 0.04, respectively). These results indicate that people with higher calcium levels tend to fall asleep easier and have shorter total sleep time. On the other hand, in non-shift workers, those significant correlations disappeared, but there was a negative correlation between calcium and phase (Figure 6A,B: r = −0.20, *p* = 0.0028; r = −0.18, *p* = 0.03). This result shows that higher calcium levels are related to earlier acrophase of activity in non-shift workers, which indicates their maximal activity present earlier during the day.

In Figure 4, although vitamin D levels positively correlated with calcium levels in both groups, vitamin D levels did not correlate with actigraphy-based sleep or activity parameters in shift workers. However, higher vitamin D levels were related to morning preference (higher MEQ) in non-shift workers. In addition, the results can easily have understood that sleep latency positively correlates with WASO and negatively correlates with SE in both shift and non-shift workers. However, there were positive correlations between sleep latency rest–activity rhythm parameters (phase, fit, amplitude, and SD) and negative correlation with the Connor–Davidson resilience scale in shift workers and two parameters (mean and intercept) in both groups.

We performed a correlation analysis to find the relationship between serum vitamin D and calcium levels with PSQI components (Table 3 and Table 4). Our PSQI findings were based on self-questionnaires and had seven components; each part evaluates specific factors of sleep quality. For example, PSQI 1 complex indicates how subjectively they feel about their sleep quality. PSQI 2–3 complex assesses sleep latency and duration, respectively. Furthermore, 4 complex means habitual sleep efficiency, 5 evaluates sleep disturbances, 6 evaluates the use of sleep medicine, and 7 indicates daytime dysfunction. As a result, PSQI components 6 and 7, which indicate the use of sleep medicine and daytime dysfunction, showed a negative correlation with vitamin D and calcium levels in shift workers. These results mean that higher calcium and vitamin D levels are related to lower sleep medicine usage and less risk of daytime dysfunction. Otherwise, there was no significant correlation between PSQI complex and calcium and vitamin D levels in non-shift workers. 

## 4. Discussion

In our study, we found that shift workers had loser serum calcium levels with more disturbed sleep and rest–activity rhythmicity. The shift workers showed longer WASO time, indicating difficulty sustaining sleep, lower sleep efficiency, and longer total sleep time than non-shift workers. Moreover, delayed phase and lower rhythmicity (lower fit and amplitude) were found in the shift workers. In clinical parameters using self-questionnaires, subjective poor sleep quality by PSQI, daytime sleepiness by ESS, anxiety and depression by HADS, fatigue levels by FSS, and eveningness by MEQ were significantly exacerbated in the shift workers.

### 4.1. Rest–Activity Rhythm Measured by Actigraphy

Actigraphy is a non-invasive way to monitor the cycle of rest and activity in humans. Actigraphy is a small wearable device that measures sleep–wake patterns for a few weeks while worn on the wrist like a watch [34]. After converting motion into an analog electrical form, it is stored in a digital format. The actigraphy data can be interpreted with some additional information such as light and sleep diary [35]. Sleep parameters such as time in bed, sleep onset, total sleep time, sleep efficiency, and wake after sleep onset can be calculated from the activity data. In addition, rest–activity rhythm can be also analyzed by cosigner analysis for calculating acrophase and amplitude of the rhythm.

### 4.2. No Statistical Difference in Vitamin D Levels between Shift and Non-Shift Workers

Previous studies have suggested that night or shift workers have lower vitamin D levels than daytime workers [8,9]. In our results, although serum vitamin D levels were low in shift workers, there was no statistical significance, consistent with a recent study [31]. Because vitamin D is synthesized by UVB from sunlight, the serum vitamin D level fluctuates widely according to the season, outdoor activity, sex, etc. Therefore, these variations in serum vitamin D levels and differences in statistical analysis methods could explain the controversial results. One study showed no difference in serum vitamin D between three groups: fixed daytime workers, rotating shift workers without night, and rotating shift workers with the night [36]. In addition, the rotating night shift group was not different from the indoor and outdoor workers, although the permanent night workers had significantly decreased vitamin D levels [37]. Furthermore, another study reported that the vitamin D level differed in male subjects according to work status (shift vs. non-shift), but there was no difference in women [38]. The lack of differences in vitamin D levels between the shift and non-shift workers could be partly attributed to the fact that majority of the subjects were females and different types of shifts were involved.

### 4.3. Decreased Serum Calcium Levels in Shift Workers

In contrast, our results showed that shift workers had significantly lower calcium levels and disturbed sleep and rest–activity rhythm than non-shift workers. The calcium levels, however, remained within normal limits, contrary to our assumption that shift workers have less sunlight exposure, therefore their vitamin D levels would be low. Interestingly, in our results, the average value of vitamin D levels between shift and non-shift workers was both deficiency levels (<20 ng/mL) [39], but there was no statistical difference between the two groups. However, the serum calcium level was in the normal range (8.9–10.1 mg/dL) [40] in both groups, but statistically lower in the shift workers. As expected, the vitamin D and calcium levels showed a positive correlation in both shift and non-shift workers, implying that vitamin D is crucial for serum calcium regulation. In recent studies, dietary calcium intake during the day was significantly increased in night shift workers [41] and inversely associated with serum calcium levels [42], consistent with our study that serum calcium was lower in shift workers. In addition, the circadian rhythm controls the calcium level, and the diurnal variation of serum calcium level was weakened in daytime sleep conditions [43]. Considering that shift workers have an impaired capacity to regulate calcium levels in accordance with the circadian rhythm, it might be more likely that their calcium levels would be less resilient to changes in vitamin D levels when compared to those of non-shift workers.

### 4.4. Lower Calcium Levels Associated with a Longer SL and TST in Shift Workers

We investigated correlation analysis between vitamin D and sleep parameters, but vitamin D only positively correlated with calcium. Rather, calcium level is negatively correlated with sleep-related parameters such as sleep latency and total sleep time in the shift workers. In other words, the lower calcium level may be associated with a longer sleep latency and a longer total sleep time. It seems counterintuitive that the lower calcium level can contribute to both a disadvantage and an advantage in sleep. Recent research has indicated that calcium plays a critical role in generating slow oscillations during NREM sleep [24]. Therefore, reduced calcium level may interfere with the generation of slow wave activity, necessitating a longer sleep time to dissipate the given sleep pressure during the wake period. It may be supposed that a low calcium level reduces slow-wave activity and increase total sleep time to maintain the homeostasis of slow-wave energy, which is the cumulative sum of slow-wave activity across total sleep time [44]. Given that this correlation between the calcium level and sleep parameters did not appear in non-shift workers, it is likely that the impact of calcium levels on sleep might not be strong enough to alter sleep parameters under the undisrupted rest–activity rhythm in non-shift workers. Therefore, it could be considered that shift workers are more vulnerable to calcium-dependent sleep disturbances than non-shift workers [45,46]. The shift workers may therefore be at a greater risk of calcium-dependent sleep disturbances when compared to the non-shift workers as a result of these findings. Even though vitamin D levels differ slightly between shift and non-shift workers, the changes in vitamin D were not able to affect sleep directly, but the calcium levels showed significant correlations with sleep parameters.

### 4.5. Rest–Activity Rhythm and Calcium Level in the Shift Workers

Another finding in our actigraphy-based study is a delayed phase and reduced rhythmicity (lower fit and amplitude) in the shift workers. Since the 24-h activity is averaged over several days, irregular activity in the shift workers can lower the average value and reduce the amplitude and fit by cosinor analysis. We also found a negative correlation between serum calcium level and circadian acrophase in non-shift workers, whereas this relationship was not observed in shift workers. This might be due to the forcefully delayed phase in the shift workers, implying that the impact of the shift work schedule on the circadian phase is much stronger than that of serum calcium level.

### 4.6. Poorer Subjective Sleep Quality in the Shift Workers

With self-assessment parameters, the serum calcium level was negatively correlated with the number of sleep medication use and the level of daytime dysfunction in the shift workers. This result indicates that the higher the calcium level, the fewer sleep problems. On the other hand, non-shift daytime workers did not show a correlation between serum calcium levels and PSQI components. It should be reminded that non-shift workers are more resilient to the change in serum calcium levels because they might have more favorable sleep hygiene, which can negate the negative impacts of decreased serum calcium levels. Therefore, the additive impact of lower calcium on poorer subjective sleep quality was significant only in shift workers with irregular sleep schedules.

### 4.7. Limitations

First, most subjects were female because they were recruited from hospital workers, primarily nurses. However, since there was no difference in gender ratio between shift and non-shift worker groups, we believe the comparisons between the two groups are statistically appropriate. Second, our statistical analysis did not include adjustments with covariates such as age, BMI, marital status, education, and smoking. Age is the reading covariate among them, and non-shift workers showed older age which might be a negative factor for sleep. Hence, the identified sleep problems in shift workers may not be explained by the age difference. Third, an actigraphy-based analysis is less accurate than a PSG analysis. Some abnormal data by non-compliant subjects may have been included in the analysis.

## 5. Conclusions

Our study investigated clinical, demographic, and actigraphy-based sleep and rest–activity parameters in shift and non-shift workers. Vitamin D levels were not significantly different between groups, but serum calcium levels in shift workers were lower than in non-shift workers. In addition, in shift workers only, the calcium levels were negatively correlated with total sleep time and sleep latency. It suggests that the lower serum calcium levels in shift workers may be associated with the more disturbed sleep–wake control and rest–activity rhythm, even if they are within the normal range. Therefore, close monitoring of serum calcium levels in patients with sleep disturbances might be needed in shift workers.

## Figures and Tables

**Table 1:** Demographic characteristics of shift workers and non-shift workers.

Characteristics	Shift Workers (*n* = 150)	Non-Shift Workers (*n* = 203)	*p*-Value
Sex, male, *n* (%)	11 (7.3)	18 (8.9)	0.604
Age, years	32.86 ± 0.62	40.40 ± 0.88	<0.001
Body mass index, kg/m^2^	21.12 ± 0.22	22.00 ± 0.20	<0.001
Marital status, married, *n* (%)	36 (24.0)	91 (44.8)	<0.001
Education, college graduates, *n* (%)	140 (93.3)	153 (75.3)	<0.001
Current smoking, *n* (%)	9 (6.0)	23 (11.3)	0.017
Drinking, *n* (%)	94 (62.7)	112 (55.2)	0.158
Coffee consumption, cups/day	1.12 ± 0.07	1.23 ± 0.07	0.18
Physical activity, *n* (%)	90 (60.0)	113 (55.7)	0.415
Medication use, *n* (%)	29 (16.5)	57 (23.0)	0.058
Medical comorbidities, *n* (%)	20 (13.3)	33 (16.3)	0.447

Data are presented as numbers (percentage) or mean ± standard error of means (S.E.M). The independent *t*-test and chi-square test were used for continuous and categorical variables, respectively.

**Table 2:** A comparison of collected parameters of shift and non-shift workers.

	Shift Workers (*n =* 150)	Non-Shift Workers (*n* = 203)	*p*-Value (*p* < 0.05 *)
***Laboratory tests***			
25(OH)D (ng/mL)	13.45 ± 0.46	14.58 ± 0.59	-
Calcium (mg/dL)	9.31 ± 0.03	9.38 ± 0.02	*
***Actigraphy-based sleep parameters* ^a^**			
SL, min	5.83 ± 0.31	5.53 ± 0.30	-
TST, min	406.87 ± 3.61	385.73 ± 3.37	***
WASO, min	70.94 ± 2.01	58.51 ± 1.59	***
SE, %	84.08 ± 0.39	85.84 ± 0.33	***
TIB, min	484.10 ± 7.56	441.17 ± 5.11	-
***Actigraphy-based activity parameters* ^b^**			
Phase	14.72 ± 0.49	12.08 ± 0.42	***
Fit	0.48 ± 0.02	0.59 ± 0.01	***
Amplitude	0.48 ± 0.02	0.59 ± 0.01	***
SD	612.07 ± 24.56	759.03 ± 32.77	-
Mean	1054.62 ± 55.53	1042.83 ± 52.67	-
Intercept	714.62 ± 53.53	744.96 ± 33.23	-
***Clinical parameters*** ^c^			
MEQ	42.58 ± 0.61	47.78 ± 0.59	***
ESS	9.19 ± 0.27	7.80 ± 0.22	***
HADS	12.65 ± 0.49	11.42 ± 0.38	*
PSQI	7.91 ± 0.26	6.37 ± 0.20	***
FSS	45.23 ± 1.18	36.05 ± 1.03	***
CD-RISC	62.28 ± 1.06	63.06 ± 1.07	-

Data are presented as mean ± S.E.M. * *p* < 0.05, *** *p* < 0.001 denotes statistical significance by two-sample *t*-test. ^a^ Parameters for sleep based on actigraphy. ^b^ Parameters for rest–activity rhythm based on actigraphy. The italics indicate the category titles. ^c^ Clinical parameters were collected through self-report questionnaires. *Abbreviations*: SL, sleep latency (min); TST, total sleep time (min); WASO, wake after sleep onset (min); SE, sleep efficiency (TST/TIP * 100%); TIB, time in bed (min). Phase, the time of day of the peak of the curve; Fit, the value of the correlation with the fitted curve; Amplitude, the height of the rhythm; SD, standard deviation; Mean, the mean of the rhythm (=MESOR); Intercept, the activity intensity when the time is 0. MEQ, morningness-eveningness questionnaire; ESS, Epworth sleepiness scale; HADS, hospital anxiety and depression scale; PSQI, Pittsburgh sleep quality index; FSS, fatigue severity scale; CD-RISC, Connor–Davidson resilience scale.

**Table 3:** Correlation between serum 25(OH)D levels and PSQI components.

	Serum 25-Hydroxyvitamin D Level			
PSQI Complex	Shift Workers (*n* = 150)		Non-Shift Workers (*n* = 203)	
	r	*p*-Value	r	*p*-Value
Component 1; subjective sleep quality	0.140	0.088	−0.024	0.737
Component 2; sleep latency	−0.029	0.725	0.080	0.254
Component 3; sleep duration	0.132	0.108	−0.037	0.596
Component 4; habitual sleep efficiency	0.066	0.421	0.053	0.450
Component 5; sleep disturbance	0.070	0.396	−0.023	0.744
Component 6; use of sleep medicine	−0.172	0.036 *	0.081	0.248
Component 7; daytime dysfunction	−0.200	0.014 *	0.039	0.578

Data are presented as r and *p*-value. r, Pearson’s correlation coefficient. * *p* < 0.05 denotes statistical significance.

**Table 4:** Correlation between serum calcium level and PSQI components.

	Serum Calcium Level			
PSQI Complex	Shift Workers (*n* = 150)		Non-Shift Workers (*n* = 203)	
	r	*p*-Value	r	*p*-Value
Component 1; subjective sleep quality	0.040	0.627	0.018	0.794
Component 2; sleep latency	−0.041	0.619	0.016	0.824
Component 3; sleep duration	0.004	0.959	−0.049	0.491
Component 4; habitual sleep efficiency	−0.019	0.817	0.020	0.775
Component 5; sleep disturbance	−0.017	0.834	0.020	0.780
Component 6; use of sleep medicine	−0.171	0.036 *	0.014	0.837
Component 7; daytime dysfunction	−0.201	0.014 *	−0.010	0.888

Data are presented as r and *p*-value. r, Pearson’s correlation coefficient. * *p* < 0.05 denotes statistical significance.

## Data Availability

Data are available upon reasonable request to the corresponding author.
